# Relationship between dietary fiber and all-cause mortality, cardiovascular mortality, and cardiovascular disease in patients with chronic kidney disease: a systematic review and meta-analysis

**DOI:** 10.1007/s40620-023-01808-4

**Published:** 2024-01-02

**Authors:** Wei Gai, Lihua Lin, Yuxuan Wang, Jia Bian, Yanling Tao

**Affiliations:** 1 Shenzhen Clinical Medical College, Guangzhou University of Chinese Medicine, Shenzhen, 510006 Guangdong Province China; 2 Department of Nursing, Longgang Central Hospital of Shenzhen, Shenzhen, 518116 Guangdong Province China

**Keywords:** Dietary fiber, Chronic kidney disease, Cardiovascular disease, Mortality, Meta-analysis

## Abstract

**Background:**

The potential protective effects of dietary fiber against all-cause mortality, cardiovascular mortality, and cardiovascular disease in patients with chronic kidney disease have not been definitively established. To verify this relationship, a systematic review and a meta-analysis were undertaken.

**Methods:**

PubMed, The Cochrane Library, Web of Science, Embase, ProQuest, and CINAHL were used to systematically search for prospective cohort studies that investigate the association between dietary fiber and all-cause mortality, cardiovascular mortality, and cardiovascular disease in individuals with chronic kidney disease (CKD). This search was conducted up to and including March 2023.

**Results:**

The analysis included 10 cohort studies, with a total of 19,843 patients who were followed up for 1.5–10.1 y. The results indicated a significant negative correlation between dietary fiber and all-cause mortality among patients with CKD (HR 0.80, 95% CI 0.58–0.97, *P* < 0.001). Subgroup analysis further revealed that the study population and exposure factors were significantly associated with all-cause mortality (*P* < 0.001). Increased dietary fiber intake was associated with a reduced risk of cardiovascular mortality (HR 0.78; 95% CI 0.67–0.90) and a reduced incidence of cardiovascular disease (HR 0.87; 95% CI 0.80–0.95) among patients with CKD.

**Conclusions:**

The pooled results of our meta-analysis indicated an inverse association between dietary fiber intake and all-cause mortality, cardiovascular mortality, and cardiovascular disease.

**Graphical abstract:**

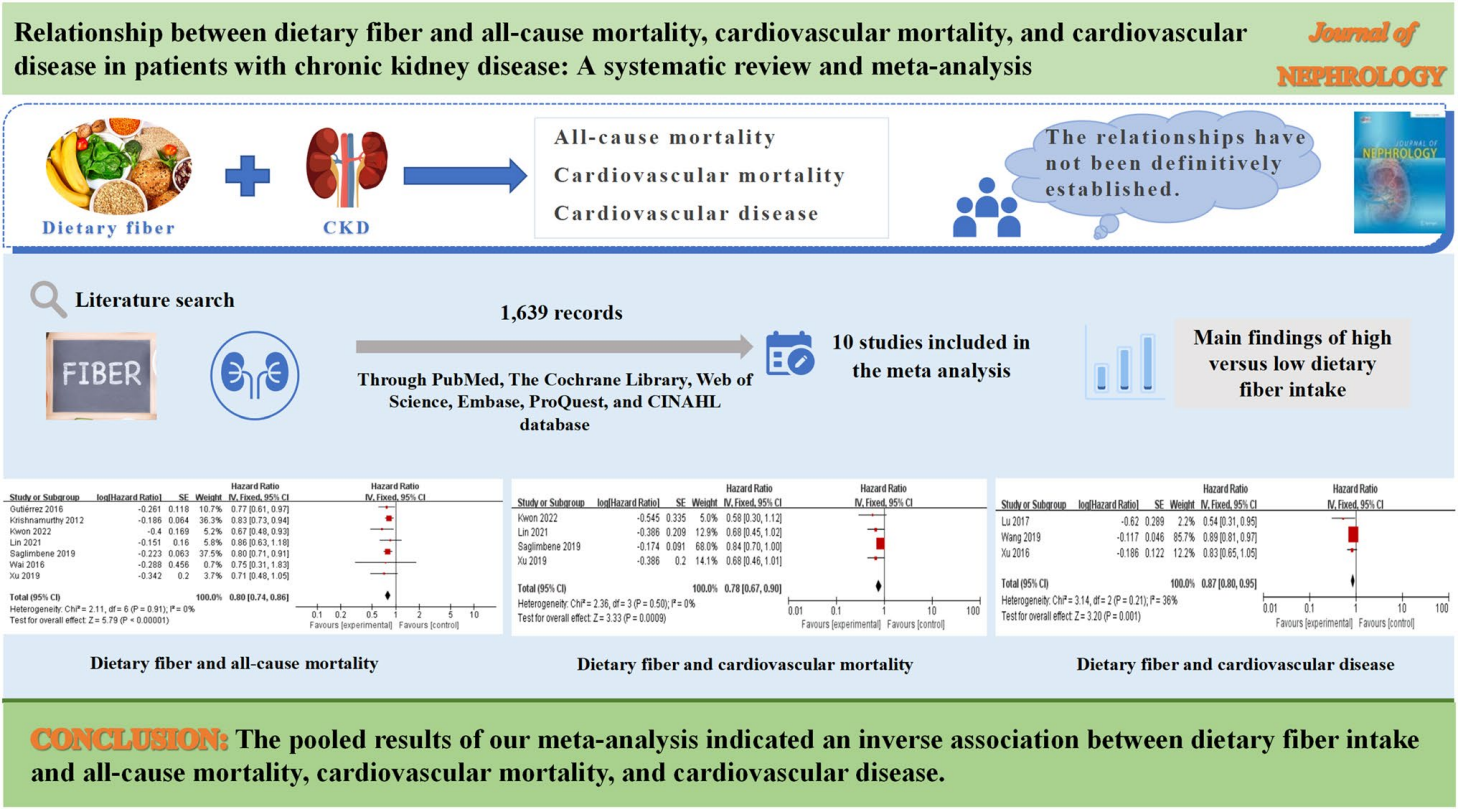

**Supplementary Information:**

The online version contains supplementary material available at 10.1007/s40620-023-01808-4.

## Introduction

Chronic kidney disease (CKD) is a progressive, incurable disease that is characterized by high morbidity, mortality, and economic burden [1]. More than 10% of the global population suffers from CKD, which amounts to about 800 million people [2]. Mortality is higher in patients with CKD than in those with healthy kidneys, and the risk exponentially increases with disease progression [3]. In 2017, global statistics showed that 1.2 million people died from CKD, and 1.4 million succumbed to cardiovascular disease that was caused by impaired kidney function [4, 5]. Chronic kidney disease is predicted to rank fifth among the leading causes of death by 2040 [6]. As renal function continuously declines, the disease eventually develops into end-stage kidney disease (ESKD). Cardiovascular events are a prevalent complication among patients who are undergoing dialysis, with reported cardiovascular mortality rates being 10–20 times higher in dialysis patients than in the general population [7]. Although dialysis technology has become increasingly sophisticated, the prognosis for ESKD remains poor. Therefore, identifying significant risk and protective factors that are associated with survival in patients with CKD is crucial.

Dietary nutrition is an essential component of CKD therapy [8]. The Kidney Disease Outcomes Quality Initiative (KDOQI) clinical practice guidelines for nutrition in CKD list healthy dietary patterns, such as the Mediterranean diet, the Dietary Approaches to Stop Hypertension (DASH) diet, and plant-based diets, that can improve clinical outcomes in chronic kidney disease [9, 10]. Consuming red and processed meat has been linked to CKD development and more risk of death, while eating fruits and vegetables can protect kidney function and lower the risk of cardiovascular disease in CKD patients [11–13]. Over the years, the dietary nutrients of CKD patients have steadily gained attention [14]. Many studies indicated that increasing dietary fiber intake reduces uremic toxins and inflammatory substances, in addition to improving the intestinal barrier in patients with CKD and dialysis [15, 16]. Dietary fiber refers to a heterogeneous group of non-digestible plant polysaccharides that are abundantly found in fruits, legumes, vegetables, and cereals [17]. Multiple studies have highlighted the potential benefits of more consumption of dietary fiber in mitigating the risk of all-cause mortality and cardiovascular disease among those affected by cancer, coronary heart disease, diabetes, and hypertension [18–23]. However, there is less scientific evidence highlighting the effects of dietary fiber consumption in improving the prognosis of CKD patients.

The currently available research findings regarding the association between dietary fiber and CKD prognosis are inconsistent. Several studies have indicated a correlation between dietary fiber intake and overall mortality among individuals with CKD [24, 25]. Two observational studies on patients undergoing dialysis reported an inverse association between high fiber intake and a reduced risk of cardiovascular death [26, 27]. On the other hand, several studies have found no statistical association between dietary fiber and the risk of cardiovascular disease in patients who were undergoing peritoneal dialysis (PD) and those with non-dialysis CKD [28–30]. Due to disparities in regions, sample sizes, exposure measurements, and adjustments in statistical models, various studies on the same topic may reach different conclusions. Because of contradictory findings, it is still not clear whether dietary fiber has the same beneficial effects in the CKD population. Therefore, both a systematic review and meta-analysis of prospective cohort studies are necessary to verify the relationship between dietary fiber with all-cause mortality, cardiovascular mortality, and cardiovascular disease in patients with CKD.

## Methods

The present review was registered with PROSPERO (2023: CRD42023412425) and conducted in accordance with the Preferred Reporting Items for Systematic Reviews and Meta-Analyses (PRISMA) guidelines [31].

### Search strategy

Systematic searching was conducted by two trained researchers on PubMed, Cochrane Library, Web of Science, Embase, ProQuest, and CINAHL from their inception to March 2023. MeSH terms and free-text searches were used to construct the search formula. The specific search formulas that were used were “Chronic Renal Insufficiencies or Chronic Kidney Failure or Dialysis,” “Dietary fiber,” “Mortality,” “Cardiovascular Diseases,” and “Cohort Studies.” The search strategy that was applied for each database is presented in Supplementary Information 1.

### Inclusion and exclusion criteria

The inclusion criteria were as follows: (1) *Population*: Patients with CKD defined by estimated glomerular filtration rate (eGFR) < 60 mL/min·1.73 m^2^, including patients who were undergoing hemodialysis (HD) and PD, aged ≥ 18 y; (2) *Exposure*: Dietary fiber intake or fiber from food sources; (3) *Comparison:* The highest and lowest intake; (4) *Outcomes:* Association between dietary fiber and all-cause mortality, cardiovascular mortality, and cardiovascular disease (the results had to include relative risk (RR) or hazard ratio (HR) and corresponding 95% confidence intervals (CIs)); (5) *Study design*: prospective cohort studies. The exclusion criteria were as follows: (1) Duplicate publication of data; (2) Data not extractable or merged; (3) Non-English language.

### Data extraction

Data extraction was independently performed by two reviewers (WG and LHL) using an electronic form to extract the following data: first author’s last name, year of publication, country, study design, follow-up time, age, sample size, study population, exposure assessment, outcomes of interest, exposure, risk ratios (HRs), 95% confidence interval, and adjusted variables.

### Quality assessment

Two reviewers (WG and LHL) independently assessed the quality of the included studies. In cases where no consensus was reached, a third reviewer (YXW) was consulted. The quality of the included articles was assessed using the Newcastle–Ottawa Scale (NOS) [32] consisting of eight items that were divided into three dimensions as follows: selection of study subjects; comparability between groups; and outcome measures. Each entry was awarded a maximum of one star, except for items that were related to comparability, which were awarded two stars. The NOS scores ranged from zero to nine stars, with the higher scores being associated with better quality. The quality ratings of the included studies ranged from five to eight stars. The quality ratings of all the included studies are listed in Table S1.

### Statistical analysis

Hazard ratios and 95% CIs were extracted from the included studies. Standard errors of HR values were estimated based on 95% CIs. Hazard ratios were log-transformed to facilitate data merging [33]. The heterogeneity within the included cohort studies was tested using Cochrane’s *Q* test. The *I*^2^ statistic *P* < 0.05 or *I*^2^ > 50% indicates significant heterogeneity among studies [34], and a random effects model was used to combine the effect sizes [33]. Sensitivity analysis was performed by sequentially excluding each included study to assess the contribution of a particular study on the overall effect [35]. Subgroup analyses were performed to evaluate the study population and exposure types in the included studies. The construction of a funnel plot was recommended to visually assess the presence of publication bias. Additionally, Egger’s test was employed to examine the asymmetry of the funnel plot. *P* ≥ 0.05 indicated a lack of publication bias, and *P* < 0.05 indicated potential publication bias [36]. Meta-analyses were conducted using the software RevMan 5.4 (Cochrane Collaboration, Oxford, UK) and Stata 17.0 (Stata Corp, College Station, TX).

## Results

### Literature search and study characteristics

**Fig. 1:**
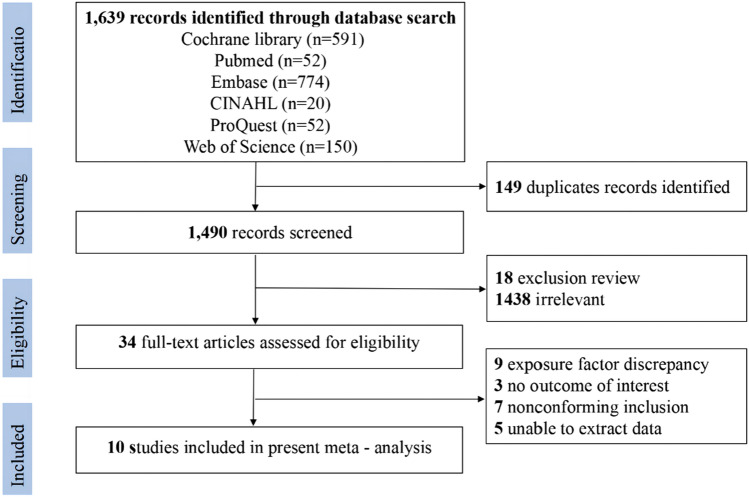
Flow diagram of the selection process of the studies

A flow diagram illustrating the selection process of the studies is presented in Fig. 1. Overall, 1639 references were first retrieved by conducting a search in an electronic database. Following the removal of duplicate articles, 1490 studies were retained. Titles and abstracts were then screened, leading to the exclusion of 1456 studies which were either reviews or were irrelevant to the current analysis. The full text of the remaining 34 studies was then read, after which 24 were excluded for various reasons, including discrepancies in exposure factors, lack of the outcome of interest, and noncompliance with the inclusion criteria. Thus, 10 cohort studies were incorporated into the meta-analysis [24–30, 37–39].

The main characteristics of the included studies are summarized in Table 1. A sum of 10 cohort studies [24–30, 37–39] that involved 19,843 patients were included in the analysis. The percentage of men in these studies ranged from 38.4% to 100%, and the duration of follow-up was 1.5–10.1 y. The included studies were conducted in different countries, namely, the United States (*n* = 2), Australia (*n* = 2), China (*n* = 4), Korea (*n* = 1), and Sweden (*n* = 1). With regard to the research population, six studies [24, 25, 29, 30, 37, 38] reported on patients with CKD, while two [26, 39] investigated HD patients, and two [27, 28] examined PD patients. Various dietary fiber evaluation tools were employed across the studies as follows: a food frequency questionnaire to measure dietary fiber intake in five studies [24, 27, 29, 38, 39]; a 24-h dietary recall questionnaire in three studies [25, 26, 37]; 3- and 7-day dietary records in two studies [28, 30]. It is also important to note that among the included studies, three examined vegetables and fruits as exposure factors, one [37] focused on vegetables, fruits, and dietary fiber as exposure factors, while two other studies [38, 39] considered vegetables and fruits as exposure factors. Dietary fiber intake was the exposure factor in seven of the studies [24–30]. Most studies adjusted for potential confounding factors such as age, sex, smoking, comorbidities, physical activity, and energy intake.

**Table 1:** The basic information of the included studies

Author	Region	Study design	Follow up (years)	Mean age (years)	Sex male (%)	Sample size	Study population	Exposure assessment	Outcomes of interest	Exposure	Quantity	HR (95% CI)	Adjustment
Gutiérrez et al. 2016 [38]	US	Prospective	6.4	68.9 ± 0.3	43	3972	CKD	The block 98 FFQ	①	A plant based diet based on fruits and vegetables	Q4 vs Q1 10.8 g/day vs 21.3 g/day	HR 0.77 (0.61–0.97)	Adjusted for age, sex, race, geographic region of residence, and energy intake, lifestyle factors ,comorbid conditions, educational achievement, annual family income, and natural log transformed urinary albumin creatinine ratio and estimated glomerular filtration rate
Krishnamurthy et al. 2012 [25]	US	Prospective	6.5	45.0 ± 15.8	48	1105	CKD	24-h dietary recall	①	Total dietary fiber	< 14.5 g/day vs ≥ 14.6 g/day	HR 0.81 (0.75–0.94)	Adjusted for age, gender, race, smoking, alcohol, leisure-time physical inactivity, and calorie intake and protein intakes
Kwon et al. 2022 [24]	Korea	Prospective	10.1	62.9 ± 8.3	38.4	3892	CKD	FFQ	①②	Dietary fiber	Q5 vs Q1 0.5–3.01 g/day vs 6.77–27.6 g/day	① HR 0.67 (0.48–0.93) ② HR 0.58 (0.30–1.11)	Adjusted for age, sex, race, geographic region of residence, and energy intake, lifestyle factors, comorbid conditions, educational achievement, annual family income, urinary albumin creatinine ratio and estimated glomerular filtration rate
Lin et al. 2021 [26]	China	Prospective	3.83	55 ± 15	59	1004	WHD	A 24 h dietary recall	①②	Dietary fiber	T3 vs T1 > 0.18(g/kg per day) vs < 0.13(g/kg per day)	① HR 0.86 (0.63–1.18) ② HR 0.68 (0.45–1.02)	Adjusted for dialysis centre, age, sex, education level, smoking status, alcohol consumption, dietary energy intake, dietary protein intake, BMI, waist: hip ratio, albumin, cholesterol, creative protein (log-transformed), creatinine, dialysis duration, Kt:V ratio and history of hypertension, diabetes and CVD
Saglimbene et al. 2019 [39]	Australia	Prospective	2.7	63 ± 15	58	8078	MHD	FFQ	①②	Fruit and vegetables	T3 (> 10 servings per week) vs T1 (0–5.5 servings per week)	① HR 0.80 (0.71–0.91) ② HR 0.84 (0.70–1.00)	Adjusted for country, age, sex, smoking, daily physical activity, myocardial infarction, vascular access type, body mass index, albumin, Charlson Comorbidity Index score, haemoglobin, and energy intake
Wai et al. 2016 [37]	Australia	Prospective	3	71.6 ± 12	59	145	CKD	A 24 h dietary recall and DHQ	①	Fiber intake; fruits and vegetables	High DHQ Score (≥ 3) vs Low DHQ Score (< 3)	Fiber intake HR 0.75 (0.31–1.85) Fruits and vegetables HR 0.35 (0.15–0.83)	Adjusted for age, gender, and eGFR, body mass index, malnutrition status (subjective global assessment), diabetes, and number of comorbidities
Xu et al. 2019 [28]	China	Prospective	3.75	57.7 ± 14.8	49.3	881	PD	The 3d dietary records	①②	Dietary fiber	T3 (> 8.3 g/d) vs T1 (< 6.2 g/d)	① HR 0.71 (0.48–1.05) ② HR 0.68 (0.37–1.26)	Adjusted for age, sex, BMI, MAP, Hb, albumin, hs-CRP, TAG, iPTH, RRF, total protein intake, total energy intake
Lu et al. 2017 [29]	China	Prospective	1.5	47.5 ± 13.2	47.1	157	CKD	FFQ	③	Dietary fiber	< 25 g/d vs > 25 g/d	HR 0.538 (0.305–0.947)	Cox regression was not performed because the study sample size was inadequate
Wang et al. 2019 [27]	China	Prospective	4	18–75^a^	49.5	219	PD	FFQ	③	Dietary fiber	T3 (> 5.88 g/d) vs T1 (< 3.71 g/d)	HR 0.89 (0.81–0.97)	Adjusted for age, gender, residual glomerular filtration rate, high sensitivity C-reactive protein, interleukin-6, daily energy intake, and protein intake and haemoglobin
Xu et al. 2016 [30]	Sweden	Prospective	9.1	70–71^a^	100	390	CKD	7-day dietary records	③	Dietary fiber	per standard deviation 3.98 g/day higher	HR 0.83 (0.65–1.05)	Adjusted for age, BMI, smoking status, physical activity, hypertension, diabetes, hyperlipidemia, eGFR, UAER, total energy intake, sodium, potassium and saturated fatty acid intake

*CKD* chronic kidney disease, *MHD* maintenance hemodialysis, *ESRD* end stage renal disease, *PD* peritoneal dialysis, *FFQ* food frequency questionnaire, *DHQ* heart wise dietary habits questionnaire, *BMI* body mass index, *Kt:V* Kt represents the effective urea clearance and duration of dialysis, and V represents the volume of distribution of urea in the body, *eGFR* estimated glomerular filtration

rate, *MAP* mean arterial pressure, *Hb* hemoglobin, *CRP* C-reactive protein, *hs-CRP* high-sensitive C-reactive protein, *TAG* Triglycerides, *iPTH* intact parathyroid hormone, *RRF* residual renal function, *UAER* urine albumin excretion rate, *HR* hazard ratio, *CI* confidence interval, *Q* quartile, *T* tertile

^a^Age range was given in the published article

① All-cause mortality ② Cardiovascular mortality ③ Cardiovascular diseases

### Dietary fiber with all-cause mortality

**Fig. 2:**
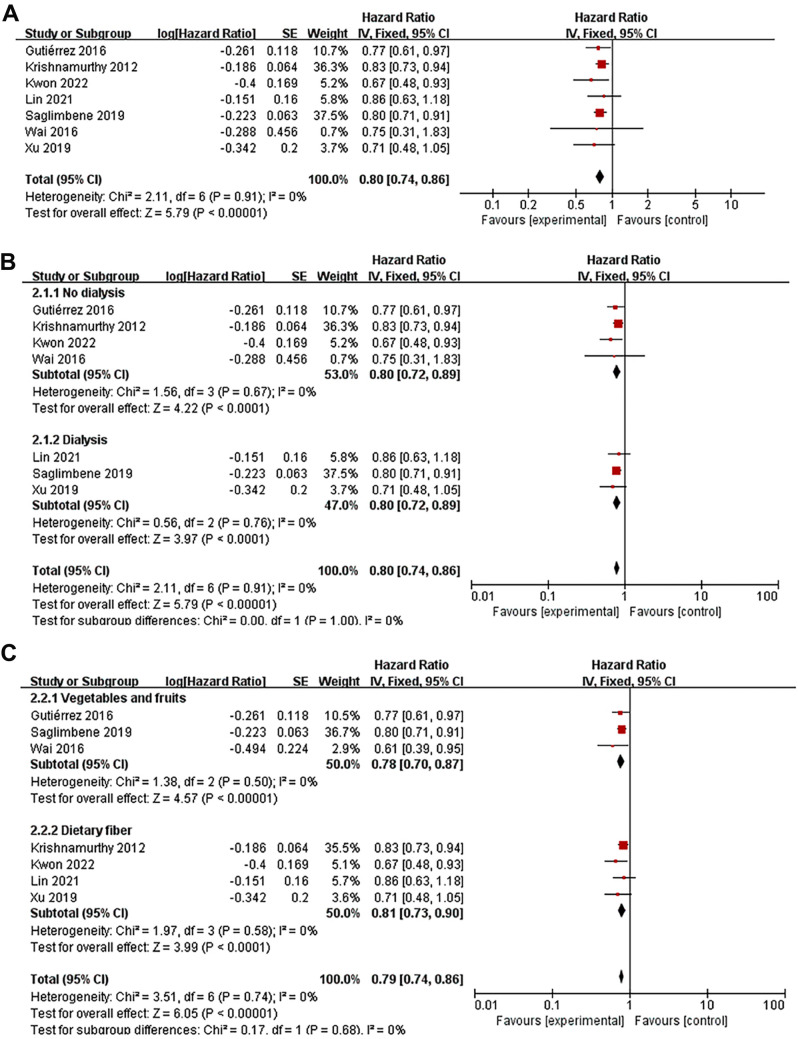
Forest plot depicting the results of a meta-analysis on the association between dietary fiber and all-cause mortality. **a** Overall meta-analysis. **b** Subgroup analysis according to study population. **c** Subgroup analysis according to dietary fiber types.

Seven studies [24–26, 28, 37–39] investigated the relationship between dietary fiber intake and all-cause mortality in CKD patients. These studies encompassed 19,086 patients, of whom 4,910 had fatal outcomes. Four studies [24, 25, 37, 38] focused on CKD patients, while three [26, 28, 39], centered on patients undergoing dialysis. Higher dietary fiber intake correlated with lower all-cause mortality in patients with CKD (HR 0.80; 95% CI 0.74–0.86, *P* < 0.001, Fig. 2A) in a fixed effects model, with no significant heterogeneity across the studies (*I*^*2*^ = 0%, *P* = 0.91). Sensitivity analysis was conducted by excluding one study at a time based on the leave-one-out method (HR 0.78–0.81, *P* < 0.001), and the results remained consistent. In a subgroup analysis segmented by the study population, both non-dialysis and dialysis patients exhibited a notable inverse correlation between dietary fiber intake and all-cause mortality (HR 0.80; 95% CI 0.72–0.98, *P* < 0.001, Fig. 2B). When examining exposure factors in a subgroup analysis, the pooled results remained in line with the primary outcomes (HR 0.78; 95% CI 0.70–0. 87, *P* < 0.001, Fig. 2C).

### Dietary fiber with cardiovascular mortality

**Fig. 3:**
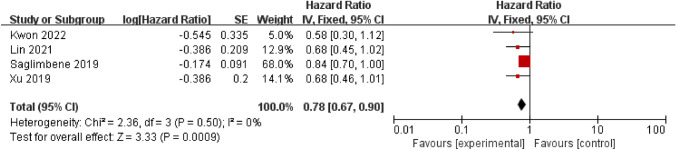
The association between dietary fiber and cardiovascular mortality

Four studies [24, 26, 28, 39] assessed the association between dietary fiber and cardiovascular mortality. A total of 13,864 patients were analyzed, resulting in 1,491 cardiovascular fatalities, three of which were dialysis patients [26, 28, 39] and one was a CKD patient [24]. Higher dietary fiber intake was associated with lower cardiovascular mortality in patients with CKD (HR 0.78; 95% CI 0.67–0.90, *P* < 0.001, Fig. 3) and no significant heterogeneity was observed across studies (*I*^*2*^ = 0%, *P* = 0.50). The sensitivity analysis revealed no changes in the result when individually excluded from the analysis (HR 0.66–0.80, *P* < 0.05).

### Dietary fiber with cardiovascular disease

**Fig. 4:**
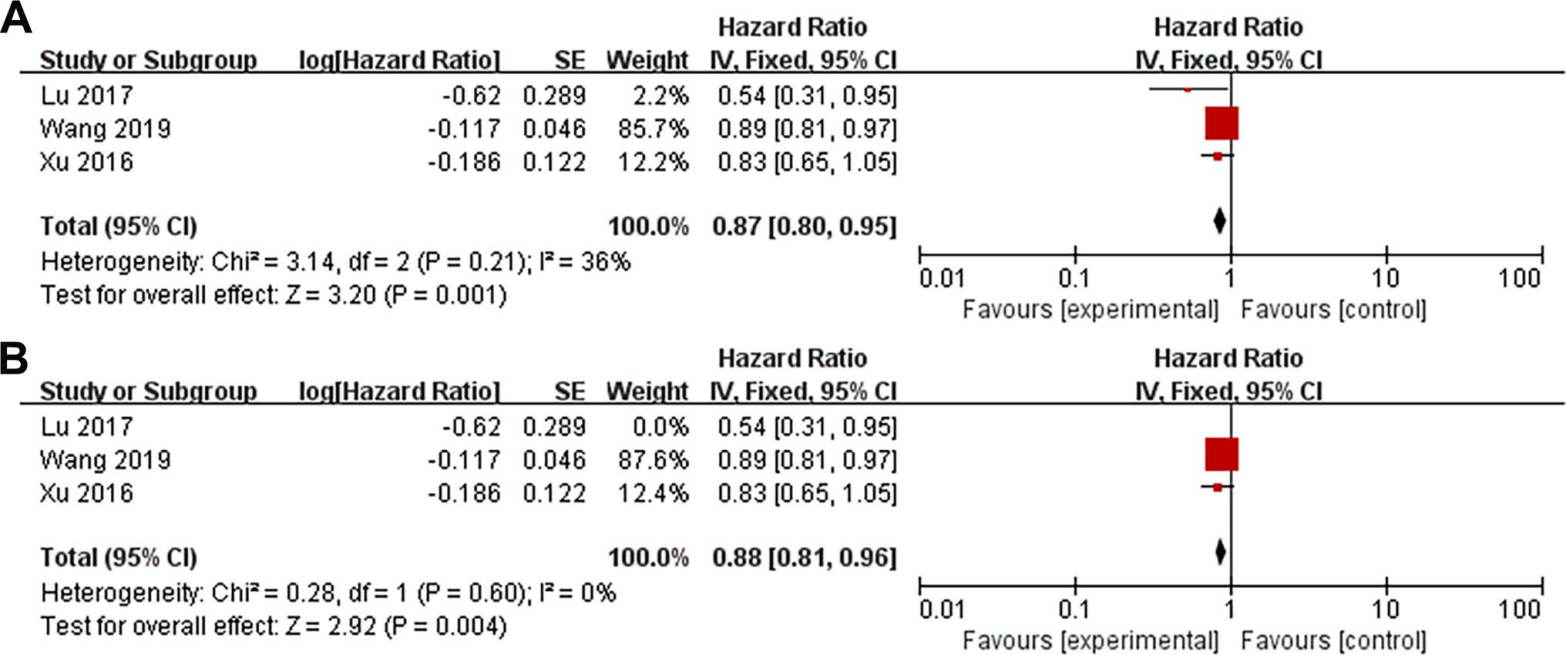
Forest plot of a meta-analysis on the relationship between dietary fiber and cardiovascular disease. **a** Overall meta-analysis. **b** Sensitivity analysis

Three studies [27, 29, 30] investigated the association between dietary fiber intake and cardiovascular disease in patients with CKD. A total of 766 patients were assessed, with 339 cardiovascular events. Two of the studies involved patients with CKD [29, 30], while one study involved patients with peritoneal dialysis [27]. Compared with higher dietary fiber intake, lower dietary fiber consumption was associated with a higher risk of cardiovascular disease in patients with CKD (HR 0.87; 95%CI 0.80–0.95, *P* < 0.05; Fig. 4A), with relatively small heterogeneity across studies (*I*^*2*^ = 36%, *P* = 0.21). The sensitivity analysis also showed no alterations in the direction of the pooled effect size. In addition, excluding the study conducted by Lu et al. [29] significantly decreased the overall heterogeneity (*I*^*2*^ = 0%, *P* = 0.60, Fig. 4B). The heterogeneity might be due to its small sample size, short follow-up time, and lack of adjustment for confounding factors.

### Publication bias

**Fig.5:**
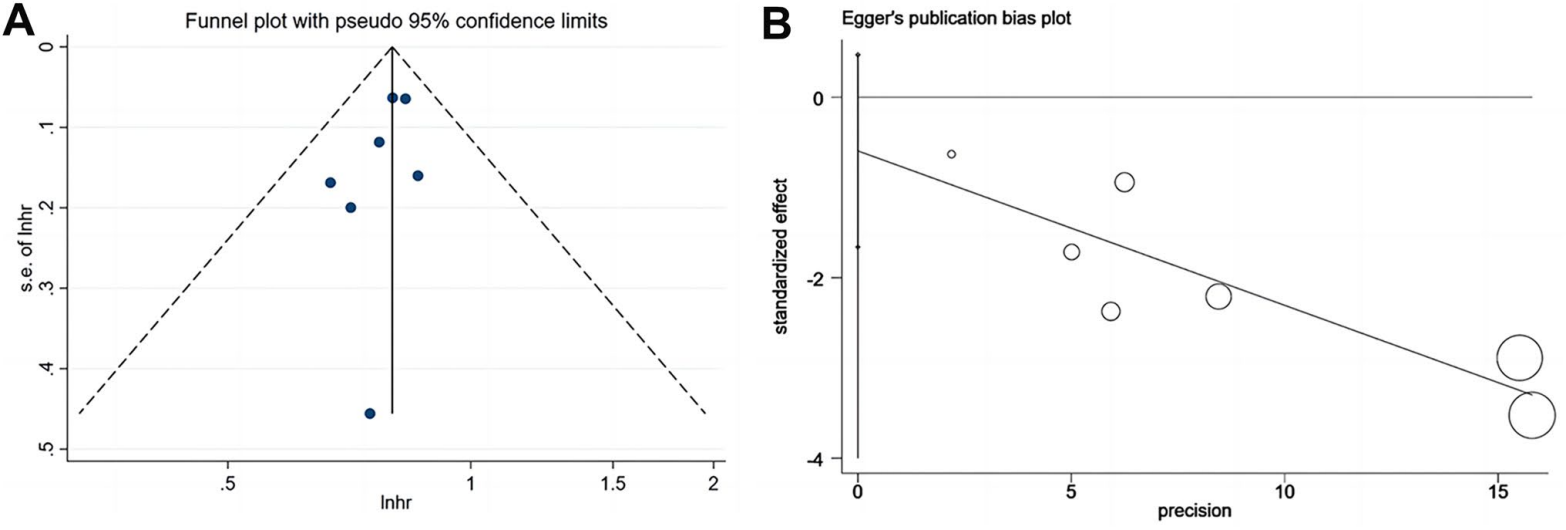
Funnel plot of the associations between dietary fiber and all-cause mortality. **a** The funnel plot with pseudo 95% confidence intervals (CIs). **b** Egger’s publication bias plot

A visual inspection of the funnel plot (Fig. 5) was combined with the results from the statistical Egger’s test in a bid to assess the possibility of publication bias. No publication bias was indicated in the relationship between dietary fiber and all-cause mortality (*p* = 0.211).

## Discussion

To the best of our knowledge, this is the first meta-analysis to show a link between dietary fiber and all-cause mortality, cardiovascular mortality, and cardiovascular disease in CKD patients. The meta-analysis of 10 cohort studies that involved 19,843 participants indicated that increasing dietary fiber consumption reduces all-cause mortality, cardiovascular mortality, and risk of cardiovascular events in CKD patients. Therefore, we hypothesize that dietary fiber may be one of the factors that influence the prognosis of CKD patients.

Several possible mechanisms have been proposed to explain the notion that dietary fiber intake reduces the risk of mortality and cardiovascular disease in CKD patients. First, serum inflammatory biomarkers are regarded as significant predictors of cardiovascular disease and mortality in CKD patients [15, 23]. The findings of an intervention trial showed a significant reduction in levels of C-reactive protein, interleukin-6, interleukin-8, and tumor necrosis factor-α, following a 6-week period of dietary fiber supplementation at doses of 10 g and 20 g per day, among those undergoing hemodialysis [40]. Dietary fiber may enhance cardiovascular health by inhibiting vascular inflammation. Second, dietary fiber decreases cardiovascular disease and death in CKD and dialysis patients by reducing intestinal uremic toxin molecules [41, 42]. Sirich et al. demonstrated that after six weeks of dietary fiber supplementation in hemodialysis patients, plasma indole sulfate, p-cresol sulfate, and plasma urea nitrogen levels significantly reduced in the fiber group [43]. These substances are strongly associated with the risk of all-cause mortality and cardiovascular disease in patients with CKD and dialysis [44, 45]. Finally, dietary fiber influences cardiovascular and metabolic problems by increasing the formation of short-chain fatty acids, in addition to modifying lipid and glucose metabolism, thereby affecting CKD development and risk [46, 47]. Therefore, CKD patients are advised to follow a multi-fiber and vegetable-based diet to restore intestinal integrity, enhance metabolic status, prevent complications, and postpone CKD progression [48].

Individual nutrient intake may play an essential role in CKD prognosis, but it can be difficult to clinically quantify it. Some dietary patterns that favor CKD outcomes include the DASH diets and Mediterranean diets, whose main shared features include being rich in plant foods and low nutritional acid load [49, 50]. Clinical trials showed that the Mediterranean diet enhances lipid profiles, lowers cardiovascular risk, and improves oxidative stress, inflammation, and insulin resistance [51]. According to Babio et al.’s review [52], the benefits of the Mediterranean diet primarily stem from individual nutrients, such as a high intake of fiber and polyunsaturated fatty acids, along with a low intake of saturated fatty acids. The same study revealed that a fruit- and vegetable-rich alkaline diet can reduce the net acid burden in CKD patients, which may contribute to the preservation of renal function in CKD patients [53]. Although the Mediterranean diet reduces the risk of chronic disease, the fact that the Western diet involves high consumption of red meat, animal fat, pastries and desserts, and low consumption of fresh fruits and vegetables associates it with an increased risk of chronic disease [54]. Compared to non-Western dietary patterns, Western dietary patterns are linked with higher risks of inflammation, cardiovascular disease, and mortality [55, 56], as well as amplification of effects on the incidence and progression of kidney disease.

Although there was no statistical heterogeneity in the included studies, some clinical heterogeneity was noted. First, there are variations in the evaluation of exposure across studies. In the included studies, food frequency questionnaire, 24-h dietary recalls, and dietary records were primarily utilized to evaluate dietary fiber intake. In two trials, the food frequency questionnaire was used to track patients’ average consumption frequency and food consumption during a one-year period. However, the food frequency questionnaire varies by region. For instance, the food frequency questionnaire that was utilized by Chinese scholars contains 15 food categories and 118 food items [29], whereas the one that was used by Korean scholars had 103 food types [24]. Due to differences in regional dietary habits, there may be variations in food types when the questionnaire is used, and the evaluation of dietary fiber intake may also be affected by food composition. Another 24-h dietary recall method is for the researcher to ask the patient to evaluate and describe the quantity and kind of all food items that they would have eaten in the preceding 24 h, and then compute the food intake using food models, food measuring tools, or food maps. Despite the fact that the two included studies were reviewed by well-trained researchers, it is still possible to over- or underestimate dietary fiber intake due to recall bias. The other two studies included employed three-day and seven-day dietary recording methodologies [28, 30]. This approach does not rely on patients’ memories and can record food intake components, volumes, and cooking methods in real-time, but it has limitations. Participants’ compliance may decrease, resulting in a restricted assessment of dietary consumption. Second, the study population consists of non-dialysis CKD patients and dialysis patients. Aside from the variation in disease severity, patients’ nutritional needs are also varied [57]. Subgroup analysis was performed on dialysis and non-dialysis CKD patients, leading to the discovery that increasing dietary fiber intake in both CKD and dialysis patients could improve all-cause mortality. Finally, there were variations in the control of confounding variables. Due to the limited sample size, Lu et al. did not adjust for confounding factors, and some studies did not adjust for body weight and certain comorbidities [25, 27]. When the confounding factors of different studies are inconsistent, there may be a discrepancy with regard to the association between the exposure factors and outcomes, mainly due to confounder bias.

The strength of our study is that it is the first meta-analysis to indicate a link between dietary fiber and all-cause mortality, cardiovascular mortality, and cardiovascular disease in CKD and dialysis patients. However, the current study has several limitations as well that should also be considered when applying the study’s conclusions. First, studies that use food frequency questionnaires or dietary recall to assess dietary fiber intake may be susceptible to recall bias, which could reduce the validity of the study results. Second, the number of studies that were included in this study was limited. Moreover, a dose–response meta-analysis was not conducted. Third, the stage of CKD has a significant impact on mortality. Given the objective limitations, we did not undertake subgroup analysis or separate meta-analysis for the distinct stages of CKD. Future research should focus on the impact of CKD stage on the effects of dietary fiber and its association with all-cause mortality. Fourth, all the included studies were observational, and we were unable to account for the confounding factors that were inherent to the original studies. Despite the fact that the majority of studies accounted for some of the known confounding variables using statistical methods, unidentified confounding variables may still exist and be a source of bias. Finally, the limited number and sample size of the included studies may have negatively affected the study’s power. We hope that well-designed research with larger samples, along with randomized controlled trials will be conducted in the future to back up the findings from this study.

## Conclusions

In conclusion, our meta-analysis indicates, for the first time, that increasing dietary fiber consumption reduces all-cause mortality, cardiovascular mortality, and cardiovascular disease in CKD patients. This study provides a reference for future dietary fiber intake in CKD patients, and additional high-quality studies are required to support the findings that are presented in this study.

### Supplementary Information

Below is the link to the electronic supplementary material.

Supplementary file1 (DOCX 33 kb)

## Data Availability

The datasets generated during and/or analyzed during the current study are available from the corresponding author on reasonable request.
