# Supplementary material for: Relationship between dietary fiber and all-cause mortality, cardiovascular mortality, and cardiovascular disease in patients with chronic kidney disease: a systematic review and meta-analysis
Source: J Nephrol. 2024 Jan 2;37(1):77–93. doi: 10.1007/s40620-023-01808-4 (PMC10920433; doi:10.1007/s40620-023-01808-4)
Supplement: Supplementary file 1 — Supplementary file1 (DOCX 33 kb) [file 40620_2023_1808_MOESM1_ESM.docx]

**Supplementary materials**

Supplemental **Information 1** Search strategies

1. **Pubmed, Embase and Cochrane:**

**Terms for patient population**

**#1**：“Kidney Failure, Chronic [mesh term]” OR “End-Stage Kidney Disease” OR “Disease, End-Stage Kidney” OR “End Stage Kidney Disease” OR “Chronic Kidney Failure” OR “dialysis [mesh term]” OR “Dialyses” OR “Renal Dialysis [mesh term]” OR “Dialyses, Renal” OR “Hemodialysis” OR “Peritoneal Dialysis” OR “Renal Insufficiency, Chronic [mesh term]” OR “Chronic Kidney Disease” OR “Chronic Kidney Insufficiency”

**Terms for exposure**

**#2**：“dietary fibre [mesh term]” OR “Wheat Bran” OR “Fiber intake” OR “whole grain” OR “vegetables [mesh term]” OR “Fruit [mesh term]” OR “Inulin [mesh term]” OR “Cellulose [mesh term]” OR “Polyanhydroglucuronic Acid” OR “alpha Cellulose” OR “Whole Grains [mesh term]” OR “Grain, Whole” OR “ Grains, Whole” OR “Pectins [mesh term] ” OR “ Pectinic Acid” OR “ Methoxypectin” OR “Oligosaccharides [mesh term]” OR “Oligosaccharide” OR “Lignin [mesh term]” OR “Lignins”

**Terms for outcome**

**#3**：“Mortality [mesh term]” OR “Mortalities” OR “Case Fatality Rat”e OR “Case Fatality Rates” OR “Cardiovascular Diseases [mesh term]” OR “Cardiovascular Disease” OR “Disease, Cardiovascular” OR “Major Adverse Cardiac Events” OR “Cardiac Events” OR “Cardiac Event” OR “stroke [mesh term]” OR “ischemic stroke” OR “transient ischemic attack” OR “coronary heart disease [mesh term]” OR “CHD” OR “CVD” OR “heart attack” OR “myocardial infarct” OR “MI” OR “cardiomyopathy” OR “heart failure” OR “ischemic heart disease” OR “acute coronary syndrome” OR “coronary artery disease” OR “atherosclerosis” OR “arteriosclerosis”

**Terms for study design**

**#4**：“Cohort Studies [mesh term]” OR “Cohort Study” OR “Studies, Cohort” OR “Study, Cohort” OR “Concurrent Studies” OR “Studies, Concurrent” OR “Longitudinal Studies [mesh term]” OR “Longitudinal Study” OR “Studies, Longitudinal” OR “Study, Longitudinal”

**#1 AND #2 AND #3 AND #4**

1. **Web of science:**

**TS=**(Peritoneal Dialysis OR Dialyses, Peritoneal OR Peritoneal Dialyses OR Chronic Renal Insufficiencies OR Chronic Renal Insufficiency OR Chronic Kidney Insufficiency OR Chronic Kidney Disease OR End Stage Kidney Disease OR End Stage Kidney Disease OR Chronic Kidney Failure OR renal dialysis OR Renal Dialyses OR Hemodialysis OR Hemodialyses OR dialysis OR Dialyses)

**TS=**(Oligosaccharides OR Oligosaccharide OR Lignin OR Lignins OR Pectins OR Pectin OR Pectinic Acid OR Methoxypectin OR Methoxylpectin OR Whole Grains OR Whole Grain OR Whole Grain Cereals OR Fruit OR Inulin OR Cellulose OR Polyanhydroglucuronic Acid OR alpha Cellulose OR Fruit OR Inulin OR Cellulose OR alpha CelluloseOR alpha Cellulose OR vegetables OR Vegetable OR Dietary Fiber OR Dietary Fibers OR Wheat Bran OR Fiber intake)

**TS=**(Mortality OR Mortalities OR Case Fatality Rate OR Case Fatality Rates OR Rates, Case Fatality OR Cardiovascular Diseases OR Cardiovascular Disease OR Major Adverse Cardiac Events OR Cardiac Events OR Cardiac Event OR stroke OR ischemic stroke OR ischaemic stroke OR haemorrhagic stroke OR hemorrhagic stroke OR transient ischemic attack OR transient ischaemic attack OR coronary heart disease OR CHD OR CVD OR heart attack OR myocardial infarct OR MI OR coronary artery disease OR CAD OR atrial fibrillation OR cardiomyopathy OR heart failure OR ischemic heart disease OR ischaemic heart disease OR acute coronary syndrome OR coronary artery disease OR atherosclerosis OR arteriosclerosis)

**TS=**(Cohort Studies OR Cohort Study OR Concurrent Studies OR Concurrent Study OR Longitudinal Studies OR Longitudinal Study)

1. **CINAHL and ProQuest:**

**Terms for patient population**

**#1：**(Peritoneal Dialysis OR Dialyses, Peritoneal OR Dialysis, Peritoneal OR Peritoneal Dialyses OR Renal Insufficiency, Chronic OR Chronic Renal Insufficiencies OR Renal Insufficiencies, Chronic OR Chronic Renal Insufficiency OR Kidney Insufficiency, Chronic OR Chronic Kidney Insufficiency OR Chronic Kidney Disease OR Kidney Failure, Chronic OR End-Stage Kidney Disease OR Disease, End-Stage Kidney OR End Stage Kidney Disease OR Kidney Disease, End-Stage OR Chronic Kidney Failure OR renal dialysis OR Dialyses, Renal OR Renal Dialyses OR Dialysis, Renal OR Hemodialysis OR Hemodialyses OR dialysis OR Dialyses)

**Terms for exposure**

**#2**：(Oligosaccharides OR Oligosaccharide OR Lignin OR Lignins OR Pectins OR Pectin OR Pectinic Acid OR Methoxypectin OR Methoxylpectin OR Whole Grains OR Grain, Whole OR Grains, WholeOR Whole Grain OR Whole Grain Cereals OR Cereal, Whole Grain OR Fruit OR Inulin OR Cellulose OR Polyanhydroglucuronic AcidOR Acid, Polyanhydroglucuronic OR alpha-Cellulose OR alpha Cellulose OR Fruit OR Inulin OR Cellulose OR Polyanhydroglucuronic Acid OR Acid, Polyanhydroglucuronic OR alpha-Cellulose OR alpha Cellulose OR vegetables OR Vegetable OR Dietary Fiber OR Dietary Fibers OR Fibers, Dietary OR Fiber, Dietary OR Wheat Bran OR Bran, Wheat OR Fiber intake）

**Terms for outcome**

**#3：**(Mortality OR Mortalities OR Case Fatality Rate OR Case Fatality Rates OR Rate, Case Fatality OR Rates, Case Fatality OR Cardiovascular Diseases OR Cardiovascular Disease OR Disease, Cardiovascular OR Major Adverse Cardiac Events OR Cardiac Events OR Cardiac Event OR stroke OR ischemic stroke OR ischaemic stroke OR haemorrhagic stroke OR transient ischemic attack OR transient ischaemic attack OR coronary heart disease OR CHD OR CVD OR heart attack OR myocardial infarct OR MI OR coronary artery disease OR heart failure OR ischemic heart disease OR ischaemic heart disease OR acute coronary syndrome OR coronary artery disease OR atherosclerosis OR arteriosclerosis)

**Terms for study design**

**#4：**(Cohort Studies OR Cohort Study OR Studies, Cohort OR Study, Cohort OR Concurrent Studies OR Studies, Concurrent OR Concurrent Study OR Longitudinal Studies OR Longitudinal Study)

**#1 AND #2 AND #3 AND #4**

Supplemental **Information 2** PRISMA checklist

| **Section and Topic** | **Item #** | **Checklist item** | **Location where item is reported** |
| --- | --- | --- | --- |
| **TITLE** | | |  |
| Title | 1 | Identify the report as a systematic review. | 1 |
| **ABSTRACT** | | |  |
| Abstract | 2 | See the PRISMA 2020 for Abstracts checklist. | 1 |
| **INTRODUCTION** | | |  |
| Rationale | 3 | Describe the rationale for the review in the context of existing knowledge. | 2 |
| Objectives | 4 | Provide an explicit statement of the objective(s) or question(s) the review addresses. | 3 |
| **METHODS** | | |  |
| Eligibility criteria | 5 | Specify the inclusion and exclusion criteria for the review and how studies were grouped for the syntheses. | 3 |
| Information sources | 6 | Specify all databases, registers, websites, organisations, reference lists and other sources searched or consulted to identify studies. Specify the date when each source was last searched or consulted. | 3 |
| Search strategy | 7 | Present the full search strategies for all databases, registers and websites, including any filters and limits used. | 3 |
| Selection process | 8 | Specify the methods used to decide whether a study met the inclusion criteria of the review, including how many reviewers screened each record and each report retrieved, whether they worked independently, and if applicable, details of automation tools used in the process. | 4 |
| Data collection process | 9 | Specify the methods used to collect data from reports, including how many reviewers collected data from each report, whether they worked independently, any processes for obtaining or confirming data from study investigators, and if applicable, details of automation tools used in the process. | 4 |
| Data items | 10a | List and define all outcomes for which data were sought. Specify whether all results that were compatible with each outcome domain in each study were sought (e.g. for all measures, time points, analyses), and if not, the methods used to decide which results to collect. | 4 |
|  | 10b | List and define all other variables for which data were sought (e.g. participant and intervention characteristics, funding sources). Describe any assumptions made about any missing or unclear information. | 4 |
| Study risk of bias assessment | 11 | Specify the methods used to assess risk of bias in the included studies, including details of the tool(s) used, how many reviewers assessed each study and whether they worked independently, and if applicable, details of automation tools used in the process. | 5 |
| Effect measures | 12 | Specify for each outcome the effect measure(s) (e.g. risk ratio, mean difference) used in the synthesis or presentation of results. | 4-5 |
| Synthesis methods | 13a | Describe the processes used to decide which studies were eligible for each synthesis (e.g. tabulating the study intervention characteristics and comparing against the planned groups for each synthesis (item #5)). | 5 |
|  | 13b | Describe any methods required to prepare the data for presentation or synthesis, such as handling of missing summary statistics, or data conversions. | 4-5 |
|  | 13c | Describe any methods used to tabulate or visually display results of individual studies and syntheses. | 5 |
|  | 13d | Describe any methods used to synthesize results and provide a rationale for the choice(s). If meta-analysis was performed, describe the model(s), method(s) to identify the presence and extent of statistical heterogeneity, and software package(s) used. | 4 |
|  | 13e | Describe any methods used to explore possible causes of heterogeneity among study results (e.g. subgroup analysis, meta-regression). | 4-5 |
|  | 13f | Describe any sensitivity analyses conducted to assess robustness of the synthesized results. | 4-5 |
| Reporting bias assessment | 14 | Describe any methods used to assess risk of bias due to missing results in a synthesis (arising from reporting biases). | 5 |
| Certainty assessment | 15 | Describe any methods used to assess certainty (or confidence) in the body of evidence for an outcome. | 4 |
| **RESULTS** | | |  |
| Study selection | 16a | Describe the results of the search and selection process, from the number of records identified in the search to the number of studies included in the review, ideally using a flow diagram. | 5 |
|  | 16b | Cite studies that might appear to meet the inclusion criteria, but which were excluded, and explain why they were excluded. | 5 |
| Study characteristics | 17 | Cite each included study and present its characteristics. | 5 |
| Risk of bias in studies | 18 | Present assessments of risk of bias for each included study. | 8 |
| Results of individual studies | 19 | For all outcomes, present, for each study: (a) summary statistics for each group (where appropriate) and (b) an effect estimate and its precision (e.g. confidence/credible interval), ideally using structured tables or plots. | 6-8 |
| Results of syntheses | 20a | For each synthesis, briefly summarise the characteristics and risk of bias among contributing studies. | 6-8 |
|  | 20b | Present results of all statistical syntheses conducted. If meta-analysis was done, present for each the summary estimate and its precision (e.g. confidence/credible interval) and measures of statistical heterogeneity. If comparing groups, describe the direction of the effect. | 6-8 |
|  | 20c | Present results of all investigations of possible causes of heterogeneity among study results. | 8 |
|  | 20d | Present results of all sensitivity analyses conducted to assess the robustness of the synthesized results. | 6-8 |
| Reporting biases | 21 | Present assessments of risk of bias due to missing results (arising from reporting biases) for each synthesis assessed. | 8-9 |
| Certainty of evidence | 22 | Present assessments of certainty (or confidence) in the body of evidence for each outcome assessed. | 6-8 |
| **DISCUSSION** | | |  |
| Discussion | 23a | Provide a general interpretation of the results in the context of other evidence. | 9 |
|  | 23b | Discuss any limitations of the evidence included in the review. | 11 |
|  | 23c | Discuss any limitations of the review processes used. | 11 |
|  | 23d | Discuss implications of the results for practice, policy, and future research. | 10-11 |
| **OTHER INFORMATION** | | |  |
| Registration and protocol | 24a | Provide registration information for the review, including register name and registration number, or state that the review was not registered. | 3 |
|  | 24b | Indicate where the review protocol can be accessed, or state that a protocol was not prepared. | / |
|  | 24c | Describe and explain any amendments to information provided at registration or in the protocol. | / |
| Support | 25 | Describe sources of financial or non-financial support for the review, and the role of the funders or sponsors in the review. | 12 |
| Competing interests | 26 | Declare any competing interests of review authors. | 11 |
| Availability of data, code and other materials | 27 | Report which of the following are publicly available and where they can be found: template data collection forms; data extracted from included studies; data used for all analyses; analytic code; any other materials used in the review. | 3 |

*From:*  Page MJ, McKenzie JE, Bossuyt PM, Boutron I, Hoffmann TC, Mulrow CD, et al. The PRISMA 2020 statement: an updated guideline for reporting systematic reviews. BMJ 2021;372:n71. doi: 10.1136/bmj.n71

For more information, visit: <http://www.prisma-statement.org/>

Supplemental **Table 1** Quality assessment of the included studies

| Study | Representativeness of the exposed  cohort | Selection of the non-exposed  cohort | Ascertainment  of exposure | Outcome of  interest was not  present at start  of study | Controls for  important  factor or  additional  factors^2^ | Assessment  of outcome | Follow-up  long enough  for outcomes  to occur^3^ | Adequacy of  follow up  of cohorts^4^ | Total quality score |
| --- | --- | --- | --- | --- | --- | --- | --- | --- | --- |
| Gutiérrez et al. (2016) | ✩ | ✩ | **-** | ✩ | ✩✩ | ✩ | ✩ | - | 7 |
| Kwon et al. (2022) | ✩ | ✩ | **-** | ✩ | ✩**-** | ✩ | ✩ | - | 6 |
| Krishnamurthy et al. (2012) | ✩ | ✩ | ✩ | ✩ | ✩**-** | ✩ | ✩ | - | 7 |
| Lin et al. (2021) | ✩ | ✩ | ✩ | ✩ | ✩- | ✩ | ✩ | - | 7 |
| Saglimbene et al. (2019) | ✩ | ✩ | **-** | ✩ | ✩- | ✩ | - | ✩ | 6 |
| Wai et al. (2016） | ✩ | ✩ | ✩ | ✩ | ✩- | ✩ | ✩ | ✩ | 8 |
| Xu et al. (2019) | ✩ | ✩ | ✩ | ✩ | ✩- | ✩ | ✩ | ✩ | 8 |
| Lu et al. (2017) | ✩ | ✩ | **-** | ✩ | - - | ✩ | - | ✩ | 5 |
| Wang et al. (2019) | ✩ | ✩ | ✩ | ✩ | ✩**-** | ✩ | ✩ | ✩ | 8 |
| Xu et al. (2016) | - | ✩ | ✩ | ✩ | ✩✩ | ✩ | ✩ | ✩ | 8 |

1. A study could be awarded a maximum of one star for each item except for the item “Controls for important factor or additional factors.”
2. A maximum of 2 stars could be awarded for this item. Studies that included adjustment for age and sex received one star, and studies that included essential confounders such as age, sex, BMI, physical activity, comorbidity, and energy intake received an additional star.
3. A cohort study with a follow-up time ≥3 years was assigned one star.
4. A cohort study with a follow-up participation rate >80% was assigned one star.
